# The expression of four pyridoxal kinase (PDXK) human variants in *Drosophila* impacts on genome integrity

**DOI:** 10.1038/s41598-019-50673-4

**Published:** 2019-10-02

**Authors:** Elisa Mascolo, Anna Barile, Lorenzo Stufera Mecarelli, Noemi Amoroso, Chiara Merigliano, Arianna Massimi, Isabella Saggio, Torben Hansen, Angela Tramonti, Martino Luigi Di Salvo, Fabrizio Barbetti, Roberto Contestabile, Fiammetta Vernì

**Affiliations:** 1 Dipartimento di Biologia e Biotecnologie “C. Darwin” Sapienza Università di Roma, Piazzale Aldo Moro 5, 00185 Roma, Italy; 2 Dipartimento di Scienze Biochimiche “A. Rossi Fanelli”, Sapienza Università di Roma, Laboratory affiliated to Istituto Pasteur Italia-Fondazione Cenci Bolognetti, Piazzale Aldo Moro 5, 00185 Roma, Italy; 3 University of Southern California, Molecular and Computational Biology Department, 1050 Childs Way, Los Angeles, California 90089 USA; 4 Dipartimento di Medicina Sperimentale, Università di Roma Tor Vergata, Via Montpellier 1, 0133 Roma, Italy; 5 Nanyang Technological University, School of Biological Science, 60 Nanyang Dr, Jurong West, 637551 Singapore; 6 Novo Nordisk Foundation Center for Basic Metabolic Research, Faculty of Health and Medical Sciences, University of Copenhagen, Blegdamsvej 3B, 2200 Copenhagen, Denmark; 7 Istituto di Biologia e Patologia Molecolari, Consiglio Nazionale delle Ricerche, Piazzale Aldo Moro 5, 00185 Roma, Italy

**Keywords:** Chromosomes, Genetics

## Abstract

In eukaryotes, pyridoxal kinase (PDXK) acts in vitamin B_6_ *salvage pathway* to produce pyridoxal 5′-phosphate (PLP), the active form of the vitamin, which is implicated in numerous crucial metabolic reactions. In *Drosophila*, mutations in the *dPdxk* gene cause chromosome aberrations (CABs) and increase glucose content in larval hemolymph. Both phenotypes are rescued by the expression of the wild type human PDXK counterpart. Here we expressed, in *dPdxk*^1^ mutant flies, four PDXK human variants: three (D87H, V128I and H246Q) listed in databases, and one (A243G) found in a genetic screening in patients with diabetes. Differently from human wild type PDXK, none of the variants was able to completely rescue CABs and glucose content elicited by *dPdxk*^1^ mutation. Biochemical analysis of D87H, V128I, H246Q and A243G proteins revealed reduced catalytic activity and/or reduced affinity for PLP precursors which justify this behavior. Although these variants are rare in population and carried in heterozygous condition, our findings suggest that in certain metabolic contexts and diseases in which PLP levels are reduced, the presence of these PDXK variants could threaten genome integrity and increase cancer risk.

## Introduction

Differently from bacteria and plants which synthesize *ex novo* the active form of vitamin B_6_, pyridoxal 5′ phosphate (PLP), in other organisms PLP production relies on the salvaging activity of two enzymes: pyridoxal 5′-phosphate kinase (PDXK) and pyridoxine 5′-phosphate oxidase (PNPO). PDXK converts PLP precursors such as pyridoxal (PL), pyridoxamine (PM) and pyridoxine (PN) taken from food into PLP, PMP and PNP, respectively. PNPO catalyzes the oxidation of PMP and PNP into PLP^1^. PLP performs many functions by working as coenzyme for a wide number of enzymes which control amino acid, lipid and carbohydrate metabolism. In addition, it takes part to the synthesis and/or catabolism of certain neurotransmitters^2,3^ and behaves as antioxidant molecule by counteracting genotoxic molecules such as oxygen reactive species (ROS) and Advanced Glycation End products (AGEs)^4–6^. Furthermore, B_6_ levels of cells can also modulate the capability to respond to steroid hormones^7^. As a consequence of such a wide spectrum of functions, disorders of vitamin B_6_ metabolism have been associated to different human pathologies such as epilepsy, diabetes, autism, schizophrenia, Alzheimer, Parkinson, Down’s syndrome and cancer^8,9^. Many reports based on either *in vitro* or *in vivo* observations support the hypothesis that vitamin B_6_ would promote antiproliferative effects on cancer cells^10^. In addition, epidemiological studies reported that elevated circulating amounts of B_6_ vitamers as well as an intense consumption of vitamin B_6_-containing food correlate with a reduced incidence of several distinct tumors including colorectal cancer^11^. Furthermore, it has been reported that high expression levels of PDXK positively correlate with survival of non-small cell lung cancer (NSCLC) patients^12^. Although many studies converge towards a protective role of B_6_ in cancer, underlying molecular mechanisms are not completely understood. Recently, several lines of evidence are accumulating which pinpoint to DNA damage as a possible link between metabolism and cancer. In particular, it has been proposed that under metabolic stress conditions or in the case of reduced availability of necessary nutrients some cellular processes such as DNA acetylation/methylation, synthesis of DNA precursors and ROS production can be altered causing DNA damage which can drive cells toward cancer^13^. Vitamin B_6_ is an antioxidant molecule and plays an important role in one-carbon metabolism, a set of reactions involved in the transfer of one-carbon groups which are at the basis of amino acid and nucleotide metabolism. As a consequence, vitamin B_6_ could represent a possible candidate to mediate the cross talk between metabolism and DNA damage. In line with this hypothesis, using *Drosophila* as model system, we demonstrated that mutations in the *dPdxk* gene cause chromosome aberrations (CABs) rescued by PLP supplementation. The same effect is produced by treating wild type flies with PLP analogues such as 4-deoxypyridoxine (4-DP) or inhibitors of PLP-dependent enzymes like cycloserine and penicyllamine^14^. Besides eliciting CAB formation, *dPdxk*^1^ mutations increase the glucose content in larval hemolymph. We demonstrated that in *dPdxk*^*1*^ mutants hyperglycemia and CABs are interconnected by a cause-effect relationship, in which high glucose is largely responsible for CABs. High glucose triggers AGE formation, which through ROS production leads to CABs^14^. The role of *PDXK* in chromosome integrity maintenance has also been demonstrated in yeast showing that mutations in the *BUD*1*6* gene, the *PDXK* ortholog, cause gross chromosome rearrangements largely mediated by altered DNA synthesis^15^. The impact of low PLP levels on genome integrity has also been tested on human cells. HeLa cells deprived of PLP by RNA interference directed against the *PDXK* gene showed chromosome aberrations (38.5% vs 2.0% in mock cells). Moreover, the treatment of mock HeLa cells with 4-DP causes chromosome aberrations^14^, p53 binding protein 1 (53BP1) and γ-H2AX repair foci accumulation^15^. Confirming the evolutionarily conserved role of *PDXK* gene in DNA integrity maintenance, we have previously demonstrated that the expression in *Drosophila* of the wild type human *PDXK* gene in a *dPdxk*^*1*^ background fully rescues CABs^14^.

In this work, we investigated whether human PDXK variants present in the population can impact on DNA integrity and be considered predictive of an increased cancer risk. For this purpose, we expressed four human PDXK variants (carrying missense mutations) into *dPdxk*^*1*^ mutant flies and tested them for CABs showing that none of them was able to completely rescue the CAB phenotype. Biochemical analysis of all these variants reveled a compromised catalytic activity and/or affinity for their substrates, which explained their “loss of function” behavior. These results translated to humans suggest that mutations in *PDXK* gene can impact on genome integrity and predispose to cancer.

## Results

### Genetic and biochemical basis of the study

We previously demonstrated that mutations in the *Drosophila dPdxk* gene cause lethality at the third larval instar and formation of CABs which can be rescued by PLP supplementation^14^. siRNAs directed against human *PDXK* gene induce the formation of CABs in fibroblasts and HeLa cells^14^ and, in addition, the PLP analogue 4-deoxypyridoxine (4-DP) increases the formation of 53BP1 repair foci in HeLa cells^15^. These findings prompted us to speculate that humans carrying mutations in the PDXK encoding gene could have an increased propensity to accumulate chromosome aberrations and a consequent increased risk to develop malignances. To evaluate this hypothesis, we decided to express human PDXK loss-of-function variants in flies homozygous for the *dPdxk*^*1*^ mutation^14^ and test them for their effects on CABs. The rationale of this strategy comes from our previous data^14^ showing that a wild type copy of the human *PDXK* gene can rescue CABs when expressed in *dPdxk*^*1*^ mutants.

**Figure 1:**
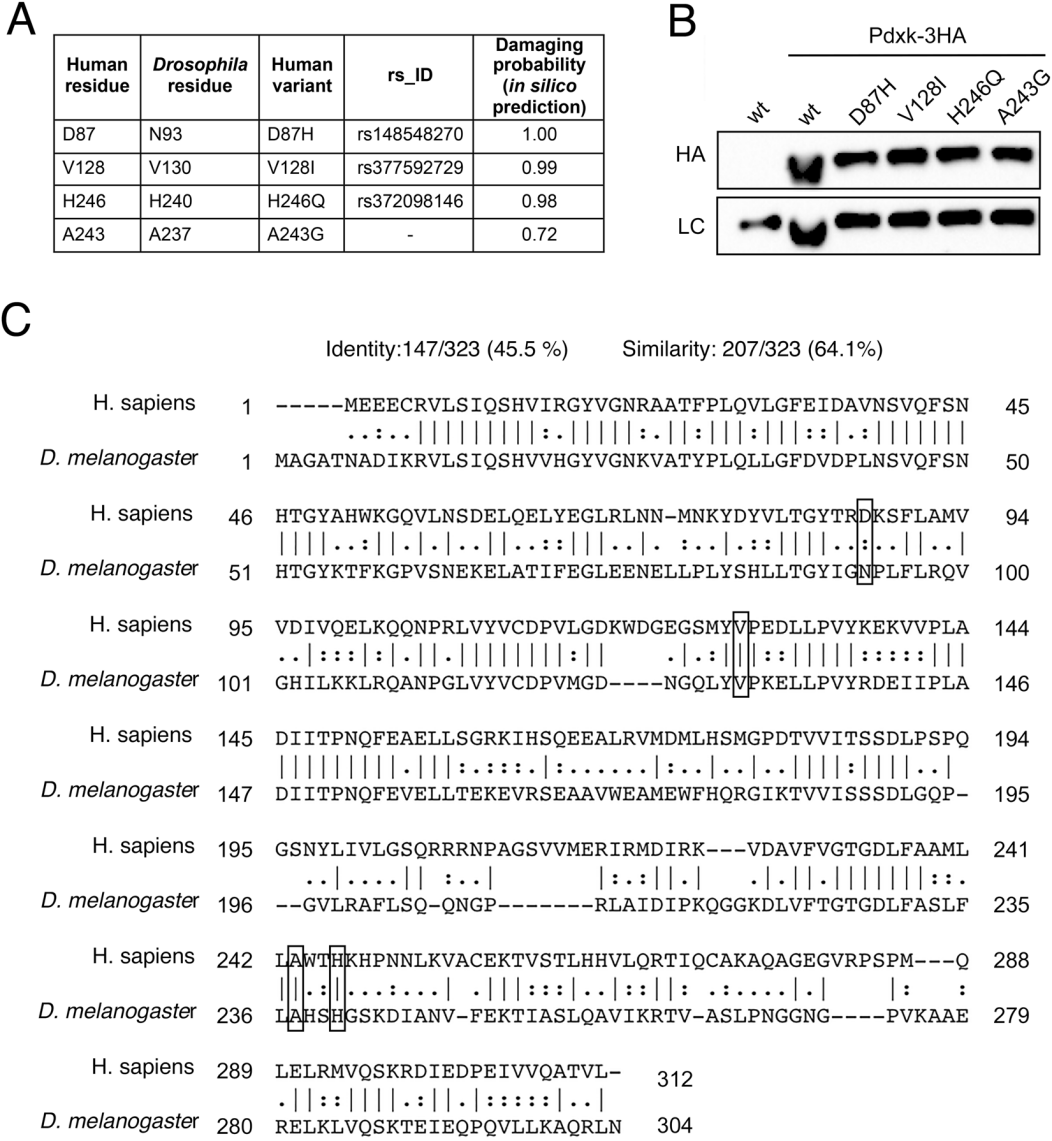
Human PDXK variants examined in this study. **(A)** Table listing human PDXK variants, studied in this work, present in Exome variant server (D87H, V128I, H246Q) or found at genetic screening (A243). **(B)** Western blot showing protein expression of PDXK human variants in brains from *dPdxk*^*1*^ *Drosophila* mutants. An antibody anti-HA was used to detect PDXK proteins. α-tubulin served as loading control (LC). **(C)** Clustal Omega pairwise sequence alignment of *Drosophila melanogaster* Pdxk (NP_996031.1) with that of Homo sapiens (NP_003672.1). Dash indicates the identical residues, colon indicates the conserved residues. Mutated residues carried by variants examined in this study are boxed.

We analyzed four human PDXK variants: Asp87His (D87H), Val128Ile (V128I), His246Gln (H246Q) and Ala243Gly (A243G). The first three have been picked up from the Exome variant server (Exome variant server, http://evs.gs.washington.edu/EVS/) which contains numerous human PDXK variants not yet associated to any disease. The variants have been chosen considering their putative damaging effects predicted *in silico* by the PolyPhen-2 software and their evolutionarily conservation of the mutated residues in *Drosophila*. In particular D87H displays the highest damaging score (1.0) and concerns a conserved position in *Drosophila* and human PDXKs; V128I and H246Q carry mutations in invariant positions (http://www.flyrnai.org/cgi-bin/DRSC_orthologs.pl) and in addition display high damaging scores (0.99 and 0.98 respectively) (Fig. 1A,C). These variants are very rare in the population (their frequency ranging from 2.84e-5 to 7.97e-6; https://gnomad.broadinstitute.org/) and are carried in heterozygous state.

A243G (Fig. 1A,C) is absent in major databases, displays a damaging score of 0.72 (Supplementary Fig. S1) and has been found in a genetic screening in patients with gestational diabetes. The rationale for the aforementioned investigation was the previously reported beneficial effect of vitamin B_6_ on gestational diabetes and its possible effect on insulin secretion in a murine model^16–18^.

None of the chosen variants concerns amino acid residues that are directly involved in substrate binding or catalysis; however, these residues are located in, or connected to, pivotal structural regions of the enzyme. PDXK is a dimer of two identical monomers. Each subunit is made by 9 α-helices and 11 β-strands that form a central β-sheet flanked by helices on both sides. Asp87 is placed on the C-terminal end of an active site loop connecting α3 helix and strand β4, which plays a crucial role in substrate binding. Tyr84, which is part of this loop, directly interacts with the B_6_ vitamer substrate stacking to its pyridine ring (Supplementary Fig. S2A) (PDB code: 3KEU and Musayev *et al*.^19^). The Val128 residue is part of another active site loop, located between strands β6 and β7 (Supplementary Fig. S2B), that closes on the active site as ATP binds to the enzyme. This loop, which is referred to as a flap in pyridoxal kinases, provides hydrogen bond interactions to the ATP β- and γ-phosphates (Tyr127, adjacent to Val128, binds to the γ-phosphate of ATP), and is believed to sequester ATP for catalysis, preventing its unproductive hydrolysis in the absence of a bound B_6_ vitamer^20–22^. Thus, variants of residues V128 and D87, which are spatially quite close to each other, are expected to affect substrate binding. On the other hand, A243 and H246, which are also close together, are located at the C-terminal end of helix α7, formed by residues 231–234, on the opposite side of the monomer with respect to the active site and therefore at a distance from it (Supplementary Fig. S2B). However, the N-terminus of this helix, which is positioned at the active site, contributes with its positive charge to binding of the PLP product of the reaction catalyzed by PDXK, stabilizing its phosphate moiety^23^. A243G and H246Q variants are predicted to have milder effects than V128I and D87H on the enzyme catalytic properties.

### Human PDXK variants fail to rescue CABs in *dPdxk*^*1*^ mutant flies

**Figure 2:**
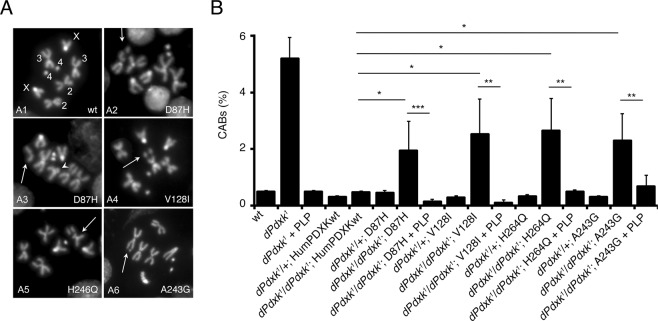
Human PDXK variants expressed in *dPdxk*^*1*^ flies do not rescue CABs. **(A)** Examples of chromosome aberrations in neuroblasts from larvae expressing PDXK variants in *dPdxk*^*1*^ background. A1 wild type metaphase; A2 and A6 autosomal chromatid deletion (arrows); A4 and A5 isochromatid deletion (arrowed); A3 isochromatid deletion at the level of centromere (arrows). Scale Bar 5 μm. **(B)** Quantification of CABs. Each bar represents the mean value ± SD obtained by scoring at least 5 brains (∼800 cells) for genotype. *^,^**^,^***Significantly different in the Student’s t test with p < 0.05, 0.01 and 0.001 respectively. (*dPdxk*^*1*^ with respect to wt and to *dPdxk*^*1*^ + PLP is <0.001, not reported in the graph).

By site-directed mutagenesis, we generated four constructs of human HA-tagged *PDXK* cDNA, each containing a variant, and introduced them in flies by germline mediated transformation. We validated the expression of these constructs by western blot analysis using an anti HA antibody (Fig 1B and Supplementary Fig. S3). By performing suitable crosses (described in Methods), we introduced these variants in *dPdxk*^*1*^ mutant flies and tested them for CABs in DAPI stained brain preparations from third instar larvae. As reported in Fig. 2A,B, the D87H, V128I, H246Q and A243G variants were unable to completely rescue CABs unless the larvae were reared in a medium containing PLP (1 mM). The expression of either wild type or variant PDXK forms in a *dPdxk*^*1*^/+ background did not produce CABs, allowing us to exclude any dominant negative effect (Fig. 1B).

Taken together, these findings suggest that all tested variants behave as loss of function alleles which impact on genome integrity.

### PDXK human variants expressed in *dPdxk*^*1*^ flies impact on glucose metabolism

**Figure 3:**
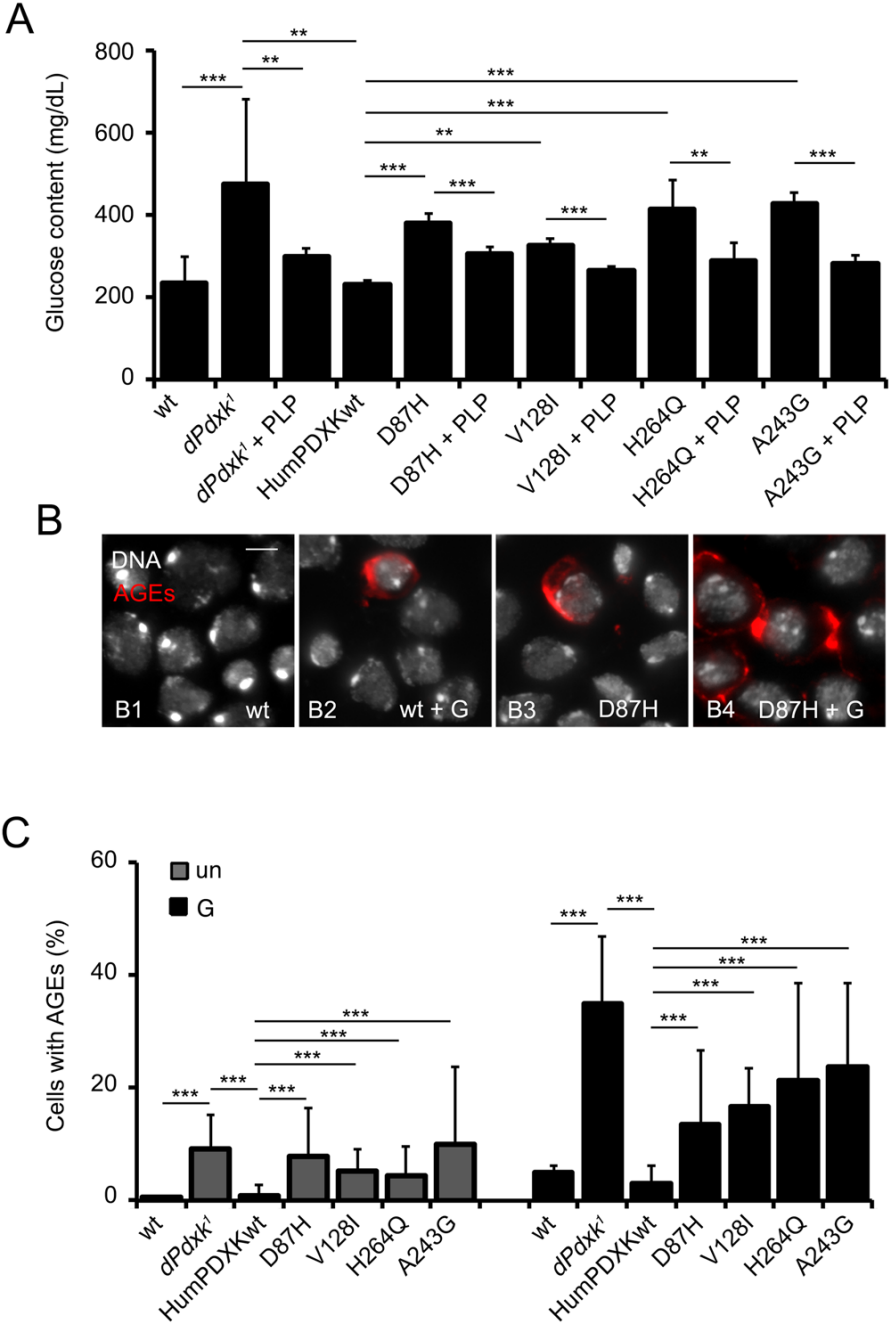
Human PDXK variants expressed in *dPdxk*^*1*^ flies do not rescue hyperglycemia. **(A)** Glucose content in hemolymph from larvae expressing either wild type or PDXK variants, reared on standard medium with or without PLP (1 mM) supplementation. Columns are the means of 5 independent sample measurements ± SD (each sample = hemolymph extracted from 20 larvae). **^,^***Significantly different in the Student’s t test with p < 0.01 and <0.001 respectively. **(B)** Examples of neuroblasts from untreated and 1% glucose treated brains expressing wt and D87H PDXK stained with an anti-human AGE antibody. Scale Bar 5 μm. G = glucose **(C)** Frequencies of AGE-positive cells in brains untreated (un) and exposed to 1% glucose (G). Bars in the graph represent the mean frequencies of AGE-positive cells (±SD) in three independent experiments by scoring at least 1000 cells in 4 brains. ***Significantly different in the Student’s t test with p < 0.001.

Mutations in the *dPdxk* gene, besides promoting CAB formation, increase glucose content in the hemolymph, generating “diabetic” flies^14^. We have previously proposed a model in which high glucose levels in the hemolymph resulting from the *dPdxk*^*1*^ mutation is largely responsible for CABs because it induces an overproduction of Advanced Glycation End products (AGEs). In turn, AGEs produce oxygen reactive species (ROS) that are well recognized for damaging DNA and leading to CAB formation^14^. To verify whether the expression of D87H, V128I, H246Q and A243G variants influenced glucose homeostasis, we tested glucose levels and found that none of the variants was able to significantly reduce hyperglycemia caused by *dPdxk*^*1*^ mutation, which was instead decreased by PLP treatment (Fig. 3A). In addition, immunostaining experiments showed that the *dPdxk*^*1*^ cells expressing D87H, V128I, H246Q and A243G variants accumulated AGEs, which were further increased by 1% glucose treatment (Fig. 3B,C), according to the hypothesis that AGEs are largely responsible for CABs in PLP depleted cells^9,14,24,25^.

### H246Q, D87H, V128I and A243G variants respond differently to PLP precursors

**Figure 4:**
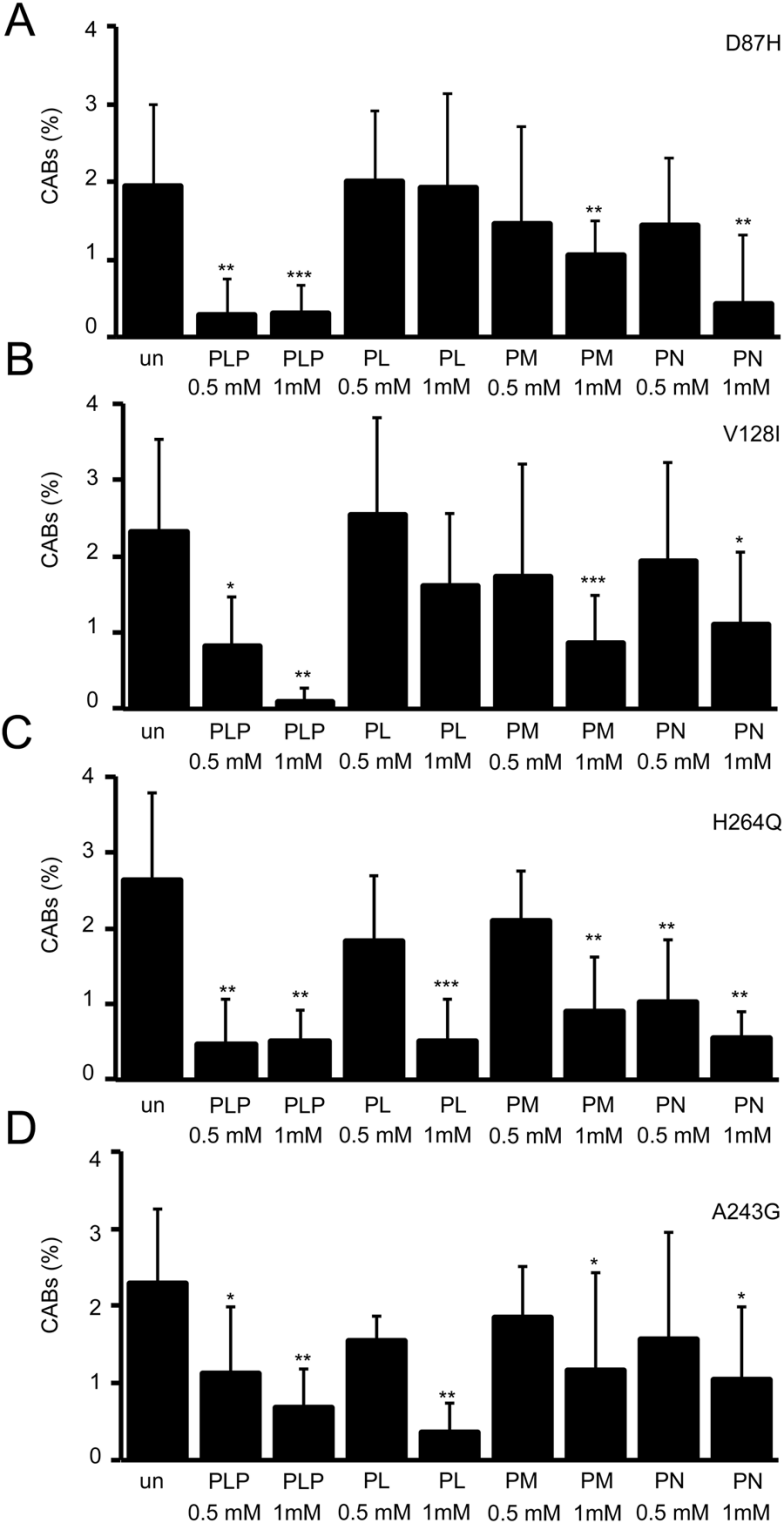
Effect of PLP precursors (PL, PM and PN) on CAB frequency in brains from larvae expressing D87H **(A)**, V128I **(B)**, H246Q **(C)** and A243G **(D)** variants. Each bar represents the mean value ± SD obtained by scoring at least 8 brains (∼1000 cells) for genotype. *^,^**^,^***Significantly different in the Student’s t test with p < 0.05, 0.01 and 0.001 respectively.

To investigate why PDXK variants failed to rescue CABs in the *dPdxk*^*1*^ background, we examined the effect of PLP precursors (PL, PM and PN) on CAB frequency in brains from larvae expressing D87H, V128I, H246Q and A243G variants. As reported in Fig. 4A–D we tested PLP precursors at two different concentrations (0.5 and 1 mM) and we found that each variant behaved differently. In brains expressing D87H, PL treatment did not rescue CABs. A feeble rescue effect was observed with PM (1 mM). In contrast, PN reduced significantly CAB frequency but only at 1 mM concentration. In brains expressing V128I, neither at 0.5 nor at 1 mM concentration PL was able to rescue CAB frequency, whereas both PM and PN rescued CABs at 1 mM concentration. In H246Q expressing cells PL and PM reduced CAB frequency only at 1 mM concentration, whereas PN reduced CAB frequency also at 0.5 mM concentration. In A243G expressing neuroblasts all precursors rescued CABs but only at 1 mM concentration. Note that PLP treatment rescued CABs at both concentrations in all tested variants.

These data suggest that the replacement of H246, V128, D87 and A243 amino acids with those found in the respective variants can impact on PDXK functionality by altering in different ways its capability to phosphorylate the substrates.

### Biochemical characterization of H246Q, D87H, V128I and A243G variants

To better understand why the four PDXK mutant proteins responded differently to PLP precursors, variant PDXK enzyme forms were recombinantly expressed in *Escherichia coli*, purified to homogeneity and characterized with respect to their catalytic properties with PL, PN and PM as substrates. As shown in Table 1, all the four mutant enzymes showed altered kinetic parameters. In particular, the D87H mutation strongly increased K_M_ for PL, and to a lesser extent also increased K_M_ for PN and PM, leaving K_M_ for ATP and k_cat_ almost unaltered. These observations are well-matched with the location of the Asp87 residue on an active site loop that plays a crucial role in the B_6_ vitamer substrate binding, and in particular with the direct interaction between the adjacent Tyr84 with the B_6_ vitamer pyridine ring (Supplementary Fig. S2A). The V128I mutation drastically increased K_M_ for PL and K_M_ for ATP with this vitamer, whereas it did not affect k_cat_. This was expected, considering the role of the active site loop containing Val128 in substrate binding and specifically in the interaction with ATP (Supplementary Fig. S2B). K_M_ for PN and PM were also increased, although with these vitamers K_M_ for ATP and k_cat_ were unaltered. This latter observation is difficult to explain on the basis of the available observed results; however, it suggests that binding of B_6_ vitamers different from PL may affect the modality of ATP binding by the loop containing Val128. The H246Q mutation somewhat reduced the affinity for ATP when using PL as substrate and the affinity for PM, while it did not affect K_M_ for PL and PN. In addition, it had the effect to halve k_cat_ with PL. Finally, A243G mutation displayed a behavior very similar to that showed by the H246Q mutation. Given the distance of Ala243 and His246 from the active site, the observed alteration of the kinetic parameters, although relatively mild, testifies that the mutation of these residues is somehow transmitted to the active site of the enzyme.

**Table 1:** Kinetic parameters of PDXK variant enzyme forms.

PDXK	PL as substrate			PN as substrate			PM as substrate		
	^a^K_M_^PL^ (µM)	^a^K_M_^ATP^ (µM)	^b^k_cat_ (min^−1^)	^a^K_M_^PN^ (µM)	^a^K_M_^ATP^ (µM)	^b^k_cat_ (min^−1^)	^a^K_M_^PM^ (µM)	^a^K_M_^ATP^ (µM)	^b^k_cat_ (min^−1^)
WT	189 ± 27	407 ± 65	58 ± 3	7.0 ± 0.9	104 ± 24	19 ± 2	5.0 ± 0.5	46.2 ± 8.2	6.9 ± 0.2
D87H	2090 ± 173	377 ± 48	40 ± 1	34.1 ± 0.4	125 ± 16	21 ± 3	26.5 ± 6.3	55.3 ± 9.1	7.2 ± 0.4
V128I	3839 ± 507	3096 ± 555	67 ± 5	24.8 ± 0.2	113 ± 6	20 ± 4	51.5 ± 6.3	56.3 ± 7.2	8.5 ± 0.2
H246Q	141 ± 10	901 ± 164	35 ± 1	11.5 ± 2.1	126 ± 1	17 ± 4	34.6 ± 7.1	76.8 ± 15.7	6.3 ± 0.3
A243G	177 ± 10	1024 ± 81	28 ± 1	9.2 ± 0.6	132 ± 23	18 ± 2	31.9 ± 4.7	34.1 ± 7.0	5.0 ± 0.1

All values are the average ± standard deviation of at least three independent determinations.

^a^Determined varying the concentration of the related substrate, while keeping the other fixed and saturating.

^b^Determined with ATP as fixed, saturating substrate. Values of k_cat_ determined with the vitamer as fixed, saturating substrate were very similar and are not reported for simplicity.

Taken together, these findings indicate that the specific changes introduced in the four examined variants reduce the PDXK functionality threatening genome integrity and impairing glucose homeostasis.

## Discussion

Limitations imposed by human subject research can be overcome by generating models of human diseases in experimental organisms. Due to widely conserved pathways which govern metabolism, *Drosophila* has been retained a precious organism for the study of metabolic human genetic diseases, either as a means of validating the causative nature of candidate genetic variants found in patients, or as a means of obtaining functional information about novel disease-linked genes.

Here we used *Drosophila* to validate the effects of four human variants of the pyridoxal kinase encoding gene on genome integrity and glucose metabolism. We previously showed that in *Drosophila* the depletion of this enzyme results in CABs which represent the consequence of hyperglycemia, another phenotype also elicited by Pdxk depletion. Also in human cells *PDXK* silencing produces CABs suggesting functional conservation^14^.

Low *PDXK* expression levels have been correlated to lung cancer^12^ and more recently also to insulin resistance which leads to type 2 diabetes^26^. However in literature, no robust data exist that link specific PDXK variants to any specific disease, except for a recent work in which two biallelic mutations in *PDXK* have been associated to polyneuropathy^27^. Conversely, numerous variants (also in homozygous condition) of the PNPO enzyme, which acts downstream of PDXK in the salvage pathway, have been associated to epilepsy^28^. The reason of this difference could be that cells with an impaired PNPO function can anyway produce some PLP throughout the conversion of PL into PLP mediated by PDXK; in contrast in cells lacking PDXK, phosphorylation of B_6_ vitamers does not occur and PLP is not produced at all. Thus, it is reasonable to expect, also by considering the wide spectrum of functions covered by PLP, that severe mutations of the *PDXK* gene seriously compromise early developmental stages causing lethality in the homozygous condition.

Databases contain PDXK variants (from heterozygous carriers), some of which carry changes in amino acid positions that are well conserved in *Drosophila*. Here we used *Drosophila* to validate the effects of four human variants (D87H, V128I, H246Q reported in the Exome variant server and the novel A243G variant) of the pyridoxal kinase encoding gene on genome integrity and glucose metabolism.

We found that none of the human variants expressed in *dPdxk*^*1*^ mutant flies could rescue CABs, differently from what observed with the wild type copy of the *PDXK* human gene^14^. This finding reinforces the notion (suggested by *in silico* analysis) that the examined variants are loss-of-function alleles. The expression of all these variants did not rescue hyperglycemia caused by *dPdxk*^*1*^ mutation nor the accumulation of AGEs that in *dPdxk*^*1*^ flies is largely responsible for CABs. The impaired rescue of hyperglycemia displayed by the A243G variant is particularly interesting because it could be considered as a preliminary indication of the association of *PDXK* gene with diabetes that will be investigated in future studies. Though the role of PDXK mutations in diabetes mellitus is purely speculative at this stage of our research, nevertheless, based on the effects of *PDXK* variants on AGEs, we favor the idea that pathophysiology of hyperglycemia might be linked to a combination of impaired insulin action on target tissues (i.e. insulin resistance) and reduced beta cell function, as it has been hypothesized for mutations of APPL1 gene associated to monogenic diabetes^29^.

The kinetic characterization of the variant enzymes showed that all mutations affected the catalytic activity of PDXK, although with different modalities (Table 1). In general, the effect of D87H and V128I mutations are more drastic than those of H246Q and A243G, according to the location of the former couple of residues in a more critical region of the enzyme. Also, it is worth noting that D87H and V128I behave similarly, and also variants H246Q and A243G display similar biochemical defects, in agreement with the relative proximity of these residues. Interestingly, PLP precursors (PL, PM and PN) have different effects on CAB frequency observed upon expression of different variants. The results obtained from the *in vitro* characterization of the enzymes parallel this observation, showing that the kinetic parameters of PLP precursors are differently affected by the mutations. In particular, the higher K_M_ for PL (about 10-fold than wild type) displayed by D87H explains why this mutant protein did not respond to PL. Differently, K_M_ values for PM and PN about 5 times higher than wild type explain the rescue observed only at 1 mM concentration.

The very high K_M_ for both ATP and PL found in the V128 mutant protein explains why PL failed to rescue CABs, whereas PN and PM reduced CAB frequency but only at the higher concentration. The H246Q variant, whose mutant enzyme form has normal kinetic parameters with PN, responds to both concentrations of this vitamer. The same mutant, displaying slightly altered kinetic parameters for ATP (when PL is used as substrate) and PM, responded positively to these precursors but only at the higher concentration. The A243G mutant enzyme displayed kinetic parameters very similar to those showed by H246Q. Similarly to H246Q, this variant responded to PL and PM; however, 0.5 mM PN was unable to reduce CAB frequency. Such different effects of PDXK mutations on the catalytic properties of PDXK are very interesting, since they are related to structure-function relationships of the enzyme. However, their full understanding is not possible on the basis of the available data and is postponed to future investigations.

PLP treatment of brains from larvae expressing each of the three variants drastically reduced CABs suggesting that this molecule can enter brain cells. A similar behavior had been previously observed in brains from either *dPdxk*^*1* ^^14^ and *sgll*^*RNAi*^ larvae (*sgll* is the *Drosophila* ortholog of PNPO)^25^. In contrast, literature reports that in humans whereas dephosphorylated B_6_ vitamers can enter cells and pass the blood-brain barrier, PLP needs to be dephosphorylated to PL before entering cells^3,30^. However, there are no data confirming that such a mechanism also occurs in flies. Thus, we can assume that at least under our experimental conditions (e.g. an excess of PLP that may force the system) PLP can enter cells without being dephosphorylated to PL.

In *Drosophila dPdxk*^*1*^ is a recessive mutation and heterozygotes do not show CABs. We could expect that the same is also true in humans; thus, heterozygous carriers of the examined variants should not exhibit CAB phenotype. However, there are certain contexts in which PLP levels are low, and reduced functionality of PDXK could be particularly critical. For example, it is known that during pregnancy an increased PLP demand to support fetal development causes a drastic reduction of PLP levels that in some cases can contribute to gestational diabetes onset. Thus, we can envisage that in pregnant women PDXK variants also carried in heterozygous condition could be dangerous. Analogously PDXK variants in heterozygous condition could also impact on genome integrity in either patients treated with drugs that reduce PLP levels or also in patients affected by pathologies such as celiac disease and diabetes which *per se* decrease PLP levels^31–33^. Thus, being able to detect PDXK mutations in such contexts could preserve genome integrity and, in the future, may pave the way for personalized cares based on B_6_ administration.

## Methods

### Materials

All reagents and buffers used for protein purification and enzyme assays were from Sigma Aldrich. The enzyme PNPOx from *E. coli* was purified as previously described in Di Salvo *et al*.^34^.

### *Drosophila* stocks and crosses

*dPdxk*^*1*^ mutation was previously described in Marzio *et al*.^14^. To introduce the transgenes carrying the PDXK variants (*PDXK* ^*VAR*^) in a mutant *dPdxk*^*1*^ background we crossed *PDXK*^*VAR*^*/CyGFP; MKRS/TM6B* females to *CyGFP/Sco; dPdxk*^*1*^*/TM6B* males.

The progeny of this cross, *PDXK*^*VAR*^ */CyGFP; dPdxk*^*1*^*/TM6B*, was crossed inter se to obtain a stable stock. From this stock larvae *PDXK*^*VAR*^*; dPdxk*^*1*^*/ dPdxk*^*1*^ selected for their non-*Tubby* phenotype have been analyzed. To test *PDXK*^*VAR*^ on a *dPdxk*^*1*^/+ background we analyzed larvae *PDXK*^*VAR*^*/Cy; dPdxk*^*1*^*/TM6B* from the same stock.

The *Oregon R* strain was used as wild-type control. All stocks were maintained and crosses were made at 25 °C on standard *Drosophila* medium (prepared from cornmeal, sucrose, brewer’s yeast, agar, water and treated with propionic acid) or on a supplemented medium (see below). The balancers and the genetic markers used in these crosses are described in detail in FlyBase (http://flybase.bio.indiana.edu/).

### Site directed mutagenesis

D87H, V128I, H246Q and A243G PDXK variants were generated by introducing mutations (by PCR based site-directed mutagenesis, QuikChange II XL Site-Directed Mutagenesis Kit, Agilent) into the wild type HA-tagged *PDXK* gene.

Primers used are:

D87H A gccaggaacgacttgtgcctcgtataacctgtg

D87H B cacaggttatacgaggcacaagtcgttcctggc

V128I A gcgaaggctcgatgtacatcccggaggacc

V128I B ggtcctccgggatgtacatcgagccttcgc

H246Q A gttattggggtgcttctgtgtccacgccagg

H246Q B cctggcgtggacacagaagcaccccaataac

A234G A cttgtgtgtccaccccaggagcatggc

A243G B gccatgctcctggggtggacacacaag

*PDXK* genes carrying the mutations were then cloned into a pCaSpeR-tubulin vector^35^. The correct generation of the variants was verified by Sanger sequencing and recombinant plasmids were introduced in flies by germline transformation (Best Gene Inc. Service, USA).

### Chromosome cytology

Colchicine-treated *Drosophila* metaphase chromosome preparations for CAB scoring were obtained as previously described in Gatti and Goldberg and in Merigliano *et al*.^24,36,37^. Anti-AGEs immunostaining of brain preparations from third instar larvae was carried out according to Bonaccorsi *et al*.^38^. Preparations were rinsed in PBS 0.1% Triton (PBST), incubated overnight at 4 °C with rabbit anti-human AGE antibody (1:200 in PBST; ab23722, Abcam,UK), rinsed in PBST, and then incubated for 1 hr at room temperature with the secondary antibody (Alexa-Fluor-555-conjugated anti-rabbit antibody 1:300 in PBST; Molecular Probes, Eugene, OR). All fixed preparations were mounted in Vectashield H-1200 with 4,6 diamidino-2-phenylindole (DAPI) (Vector Laboratories, Burlingame, CA) to stain the DNA. To quantify cells positive to AGE immunostaining at least 1000 cells were analyzed for each genotype. All cytological preparations were examined with a Carl Zeiss (Thornwood, NY) Axioplan fluorescence microscope, equipped with an HBO100W mercury lamp and a cooled charged-coupled device (CCD camera; Photometrics CoolSnap HQ).

### Treatments of larvae and isolated brains

To test the effects of PLP and PLP precursors on CABs, brains were dissected from third instar larvae and incubated for 4 hours in 2 ml of saline supplemented with 10% fetal bovine serum (FBS, Corning) with addition of 0.5 or 1 mM PLP, PM, PL or PN. 1 h before fixation brains were treated with colchicine 10^−2^ M (final concentration). To test the effects of glucose on AGE frequency brains were dissected from third instar larvae and incubated in 2 ml of saline supplemented with 10% fetal bovine serum (FBS, Corning) for 4 hours with addition of 1% glucose. Then, brains were treated according to the above described procedure for immunostaining. To test the effect of PLP on glucose content we reared flies in standard medium supplemented with PLP 1 mM. About 5 days later larvae were dissected to extract hemolymph and to measure glucose content (see paragraph below).

### Glucose measurement

Glucose concentration in hemolymph from third instar larvae was measured using the Infinity Glucose Hexokinase reagent (Thermo scientific). Hemolymph collection and glucose measurement were done as described in Marzio *et al*.^14^.

### Western blotting

Extracts for Western blotting were prepared by lysing samples of 20 brains in 150 mM NaCl, 50 mM Tris-HCl, pH 7.5, 30 mM NaF, 25 mM b-glycerophosphate, 0.2 mM Na3VO4, Triton X-100 1%, and complete Protease Inhibitor Cocktail (Roche). Extracts were immunoblotted according to Somma *et al*.^39^; blotted proteins were detected using an antibody against HA tag (Anti-HA-Peroxidase 12013819001 Roche). Anti-alpha tubulin (SIGMA) was used as loading control. Primary antibodies were detected using HRP conjugated anti-mouse and anti-rabbit IgGs and the ECL detection kit (all from GE Healthcare). Chemiluminescent blots were imaged with the ChemiDoc MP imager (Bio-Rad). Band intensities were quantified by densitometric analysis with Image Lab software (Bio-Rad).

### Production, purification and *in vitro* characterization of PDXK variant enzyme forms

Variant PDXK genes were cloned into the pET 28b(+) expression vector and this was transformed into *E. coli* Rosetta (λDE3) pLysS competent cells for protein expression. Purification of proteins was carried out as previously described in Musayev *et al*.^19^. Enzyme activity was assayed with PL and ATP (sodium salt) as substrates, using 1 µM enzyme, and were performed in a 1 cm thermostated cuvette in 100 mM NaBES buffer pH 7.3, containing 100 mM MgCl_2_, at 37 °C. The conversion of PL into PLP was followed at 388 nm as previously described in Musayev *et al*.^19^, in an Agilent 8454 UV/Vis diode array spectrophotometer. An extinction coefficient of 4900 cm^−1^ M^−1^ was used to calculate the concentration of the PLP product. Enzyme activity with PN and PM as substrates was measured in a spectrophotometric coupled assay, in which the phosphorylated products generated by PDXK were converted into PLP by *E. coli* PNPOx. The assay was carried out in the same conditions described above, with either 0.1 µM PDXK and 1 µM PNPOx (when using PN) or 0.05 µM PDXK and 5 µM PNPOx (when using PM). For each PDXK variant form, two series of initial velocity measurements were carried out, varying the concentration of one substrate while keeping the concentration of the other fixed and saturating. The obtained saturation curves were fitted to the Michaelis-Menten equation, using the software PRISM (GraphPad, La Jolla, CA, USA), obtaining estimates of the kinetic parameters.

### Statistical analysis

Results are expressed as means ± SD; probability values < 0.05 were considered statistically significant. Statistical analysis of the data was done with the two-tailed Student’s t-test.

## Supplementary information

Supplementary information

## Data Availability

The datasets generated during and/or analyzed during the current study are available from the corresponding author on reasonable request.
