# Supplementary material for: The expression of four pyridoxal kinase (PDXK) human variants in Drosophila impacts on genome integrity
Source: Sci Rep. 2019 Oct 2;9:14188. doi: 10.1038/s41598-019-50673-4 (PMC6775053; doi:10.1038/s41598-019-50673-4)
Supplement: Supplementary file 1 — Supplementary information [file 41598_2019_50673_MOESM1_ESM.docx]

**The expression of four pyridoxal kinase (PDXK) human variants in *Drosophila* impacts on genome integrity**

Elisa Mascolo^1†^, Anna Barile^2†^, Lorenzo Stufera Mecarelli^1^, Noemi Amoroso^1^, Chiara Merigliano^3^, Arianna Massimi^4^, Isabella Saggio^1,5^ , Torben Hansen^6^, Angela Tramonti^7,2^, Martino Luigi Di Salvo^2^, Fabrizio Barbetti^4^, Roberto Contestabile^2&^ and Fiammetta Vernì^1*^

^1^Dipartimento di Biologia e Biotecnologie “C. Darwin” Sapienza Università di Roma, Piazzale Aldo Moro 5, 00185 Roma, Italy

^2^Dipartimento di Scienze Biochimiche “A. Rossi Fanelli”, Sapienza Università di Roma, Laboratory affiliated to Istituto Pasteur Italia-Fondazione Cenci Bolognetti, Piazzale Aldo Moro 5, 00185 Roma, Italy

^3^University of Southern California, Molecular and Computational Biology Department, 1050 Childs Way, Los Angeles, California 90089, USA

^4^Dipartimento di Medicina Sperimentale, Università di Roma Tor Vergata, Via Montpellier 1, 0133 Roma, Italy

^5^Nanyang Technological University, School of Biological Science, 60 Nanyang Dr, Singapore 637551

^6^Novo Nordisk Foundation Center for Basic Metabolic Research, Faculty of Health and Medical Sciences, University of Copenhagen, Blegdamsvej 3B, 2200, Copenhagen, Denmark

^7^Istituto di Biologia e Patologia Molecolari, Consiglio Nazionale delle Ricerche, Piazzale Aldo Moro 5, 00185 Roma, Italy

^†^These authors contributed equally to this work

^&^Co-corresponding author

^*^Corresponding author

Fiammetta Vernì [fiammetta.verni@uniroma1.it](mailto:fiammetta.verni@uniroma1.it)

Dipartimento di Biologia e Biotecnologie “C. Darwin”, Sapienza Università di Roma

00185 Roma, Italy

Tel.+390649912471

Roberto Contestabile [roberto.constestabile@uniroma1.it](mailto:roberto.constestabile@uniroma1.it)

Dipartimento di Scienze Biochimiche “A. Rossi Fanelli”, Sapienza Università di Roma

00185 Roma, Italy

Tel. +390644427575

**Content**

**Supplementary Figure S1** Score of putative damaging effect of the A243G PDXK variant predicted by the PolyPhen-2 software.

**Supplementary Figure S2** Active site view of human pyridoxal kinase in complex with ATP and PLP

**Supplementary Figure S3** Western blot analysis for protein expression of PDXK human variants in brains from *dPdxk^1^* *Drosophila* mutants.

**
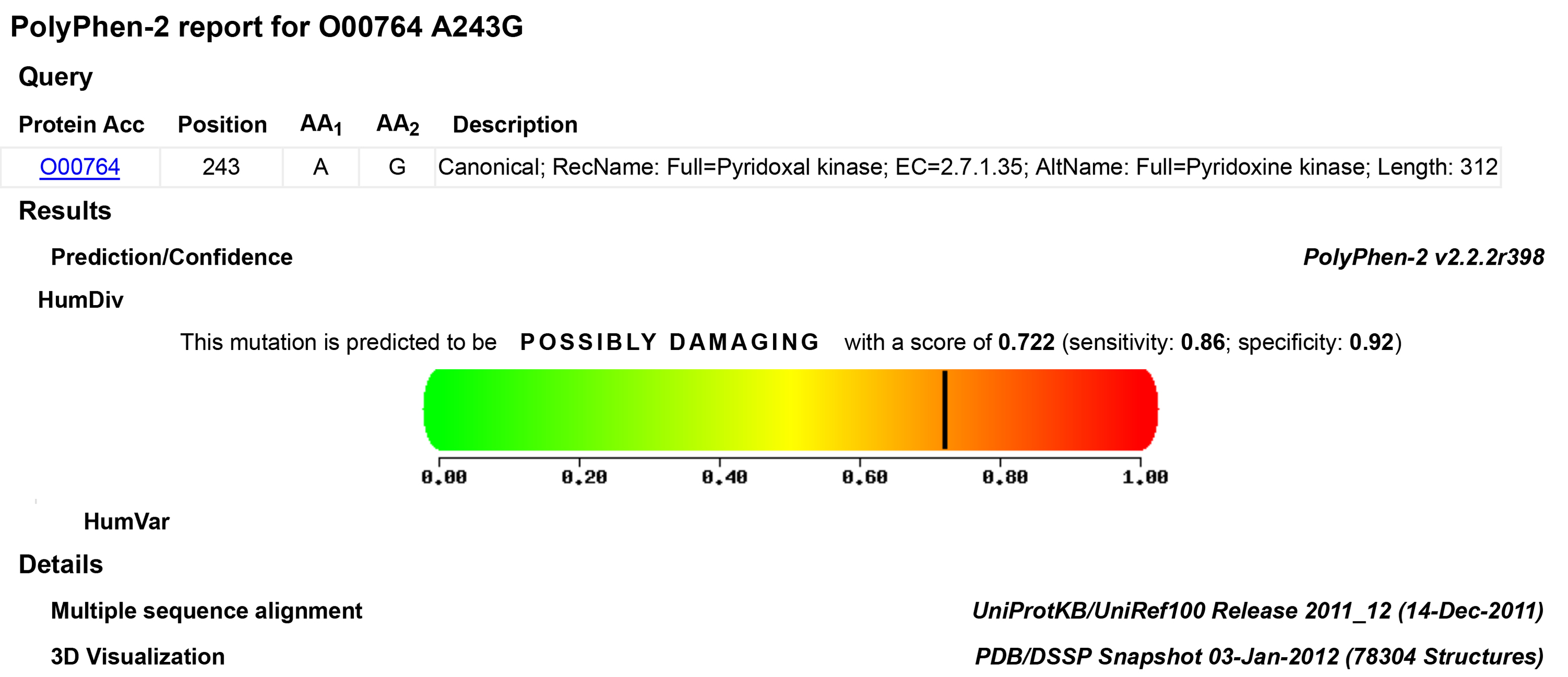
**

**Supplementary Figure S1**

Score of putative damaging effect of the A243G PDXK variant predicted by the PolyPhen-2 software (<http://genetics.bwh.harvard.edu/pph2/>)

**
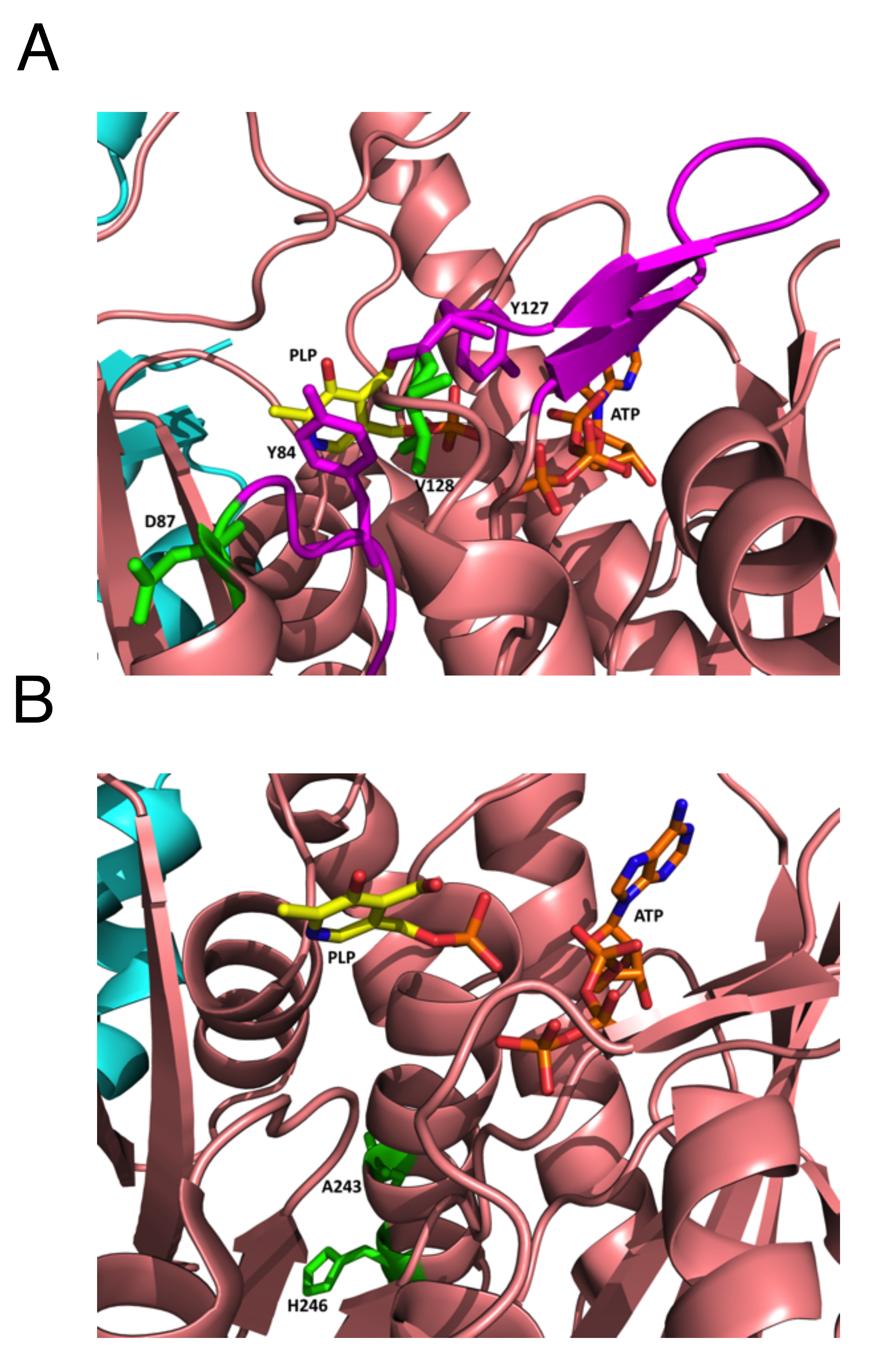
**

**Supplementary Figure S2** Active site view of human pyridoxal kinase in complex with ATP and PLP **(A)** Active site view of human pyridoxal kinase in complex with ATP and PLP (PDB code 3KEU) showing the position of residues Asp87 and Val128 (in green). The two subunits of the enzyme are shown in salmon and cyan, respectively. The crystal structure represented in this figure was obtained with both PLP and ATP bound at the active site (unpublished structure deposited in the Protein Data Bank; PDB code: 3KEU). The position occupied by PLP is believed to correspond to the site where unphosphorylated B_6_ vitamers (PL, PN and PM) also bind. PLP and ATP are in yellow and orange, respectively. The active site loops involved in binding of vitamin B_6_ (left-hand side of the figure) and ATP (right-hand side), as explained in the text, are shown in magenta. **(B)** Active site view of the same human pyridoxal kinase structure shown in panelA, from a different perspective, showing the location of residues Ala243 and His246. As explained in the text, the N-terminus of the α-helix to which Ala243 and His246 belong to is positioned at the active site and interacts with the PLP phosphate group.

**
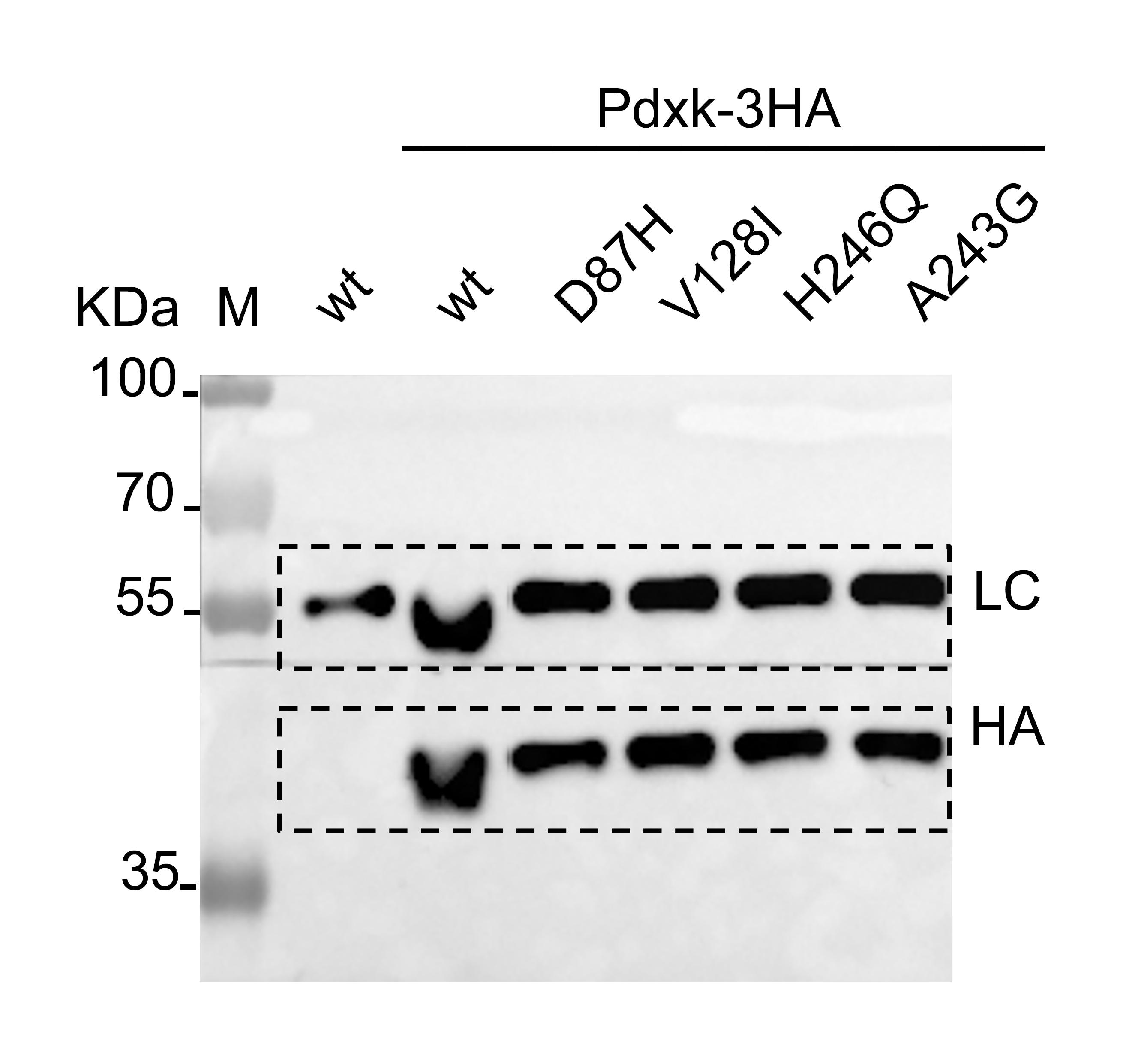
**

**Supplementary Figure S3** Western blot analysis for protein expression of PDXK human variants in brains from *dPdxk^1^* *Drosophila* mutants. Uncropped scan of the gel shown in figure 1B. Dashed boxes indicate the portion of the blot included in figure. Membrane obtained from the same gel was cut and probed with the proper antibody. α-HA was used to show the expression of PDXK-HA human variants (PDXK 36Kda + 3xHA 3KDa), α-tubulin (55 KDa) served as loading control (LC). The molecular weight of the marker (M) is indicated.
